# Efficacy and safety of praziquantel treatment against *Schistosoma mansoni* infection among pre-school age children in southern Ethiopia

**DOI:** 10.1186/s41182-023-00562-4

**Published:** 2023-12-20

**Authors:** Tafese Tadele, Ayalew Astatkie, Birkneh Tilahun Tadesse, Eyasu Makonnen, Eleni Aklillu, Solomon Mequanente Abay

**Affiliations:** 1 School of Public Health, College of Medicine and Health Sciences, Hawassa University, P.O. Box 1560, Hawassa, Ethiopia; 2 Department of Pediatrics, College of Medicine and Health Sciences, Hawassa University, P.O. Box 1560, Hawassa, Ethiopia; 3 Center for Innovative Drug Development and Therapeutic Trials for Africa, College of Health Sciences, Addis Ababa University, P.O. Box 9086, Addis Ababa, Ethiopia; 4 Department of Pharmacology and Clinical Pharmacy, College of Health Sciences, Addis Ababa University, P.O. Box 9086, Addis Ababa, Ethiopia; 5 Department of Global Public Health, Karolinska Institutet, Karolinska University Hospital, Stockholm, Sweden

**Keywords:** Efficacy, Safety, Praziquantel, Pre-school age children, Schistosomiasis, *Schistosoma mansoni*, Cure rate, Egg reduction rate, Pharmacovigilance, Ethiopia

## Abstract

**Background:**

Preventive chemotherapy with a single dose of praziquantel given to an all-at-risk population through mass drug administration is the cornerstone intervention to control and eliminate schistosomiasis as a public health problem. This intervention mainly targets school age children, and pre-school age children (pre-SAC) are excluded from receiving preventive chemotherapy, partly due to scarcity of data on praziquantel treatment outcomes.

**Methods:**

We conducted active efficacy and safety surveillance of praziquantel treatment among 240 *Schistosoma mansoni*-infected pre-SAC who received a single dose of praziquantel (40 mg/kg) in southern Ethiopia. The study outcomes were egg reduction rates (ERR) and cure rates (CRs) four weeks after treatment using the Kato–Katz technique and treatment-associated adverse events (AEs) that occurred within 8 days post-treatment.

**Results:**

The overall ERR was 93.3% (WHO reference threshold ≥ 90%), while the CR was 85.2% (95% CI = 80.0–89.5%). Baseline *S. mansoni* infection intensity was significantly associated with CRs, 100% among light infected than moderate (83.4%) or heavy (29.4%) infected children. An increase of 100 in baseline *S. mansoni* egg count per gram of stool resulted in a 26% (95% CI: 17%, 34%) reduction in the odds of cure. The incidence of experiencing at least one type of AE was 23.1% (95% CI: 18.0%, 29.0%). Stomachache, diarrhea, and nausea were the most common AEs. AEs were mild-to-moderate grade and transient. Pre-treatment moderate (ARR = 3.2, 95% CI: 1.69, 6.14) or heavy infection intensity (ARR = 6.5, 95% CI: 3.62, 11.52) was a significant predictor of AEs (*p* < 0.001). Sex, age, or soil-transmitted helminth coinfections were not significant predictors of CR or AEs.

**Conclusions:**

Single-dose praziquantel is tolerable and effective against *S. mansoni* infection among pre-SAC, and associated AEs are mostly mild-to-moderate and transient. However, the reduced CR in heavily infected and AEs in one-fourth of *S. mansoni*-infected pre-SAC underscores the need for safety and efficacy monitoring, especially in moderate-to-high infection settings. Integrating pre-SACs in the national deworming programs is recommended to accelerate the elimination of *schistosomiasis* as a public health problem.

## Background

Schistosomiasis is a common Neglected Tropical Disease (NTD) and a public health problem in the tropical and subtropical regions of Africa, Asia, the Caribbean, and South America, mainly affecting poor and rural communities [1]. Sub-Saharan Africa (SSA) is the most significantly affected continent, bearing more than 90% of the global burden of schistosomiasis, with an estimated annual death rate of 280,000 and 800 million people at risk of infection [2–4]. In SSA, intestinal schistosomiasis is mainly caused by *Schistosoma mansoni* infection, and children living in poor communities without access to safe drinking water and adequate sanitation are the most affected population. The disease can cause growth retardation, fatigue, weakness, memory impairment, anemia, poor cognition, and academic performance in infected children [4, 5]. In Ethiopia, schistosomiasis is a major public health problem causing marginalization and stigmatization, as well as a social and economic burden [6]. Schistosomiasis is highly prevalent in Ethiopia, affecting mostly preschool-aged children (pre-SAC) and school-aged children (SAC) children [7–9]. The revised NTD master plan aims to halt transmission of S. *mansoni* infection and eliminate the disease as a public health problem by 2025 [6].

For over 40 years, praziquantel (PZQ) has been the only approved drug by the World Health Organization (WHO) to treat schistosomiasis [10]. Except for the ongoing clinical trials, no approved vaccine for preventing schistosomiasis is on the market to date [11]. The WHO guideline recommends large-scale periodic administration of single-dose PZQ and albendazole combination to all at-risk populations in endemic areas through mass drug administration (MDA) as a preventive chemotherapy to control and halt transmission of *S. mansoni* and soli-transmitted helminths (STH) infections, respectively [10, 12]. In 2021, 75.3 million people received treatment for schistosomiasis in endemic areas worldwide, of which 94% of all therapy delivered globally was in the African region [13].

The MDA programs mainly target SAC and pre-SAC are excluded from deworming programs due to limited PZQ safety and efficacy information in this age group and the lack of suitable pediatric formulation [14]. Recognizing the unmet medical needs of schistosome-infected pre-SAC, a public–private partnership called Pediatric Praziquantel Consortium was formed to develop an oral dispersible tablet formulation of PZQ to treat pre-SAC infected with schistosomiasis. This Consortium supports the WHO strategic plan for schistosomiasis control and elimination in children [15]. Over a decade of annual school-based deworming with single-dose PZQ, MDA with scaled-up coverage resulted in a significant decrease in the number of disability-adjusted life years lost due to schistosomiasis [16]. However, the reduction has been less in pre-SAC mainly because they are not targeted for MDA [16]. Hence, the inclusion of pre-SAC in mass PZQ administration remains a priority to reduce the possible source of re-infection to at-risk populations covered with MDA and the occurrence of chronic schistosomiasis among pre-SAC. The safety and effectiveness of single-dose PZQ and associated factors are well-documented in SAC, but data are scarce from pre-SAC [17–20]. Few studies that assessed single-dose PZQ in pre-SAC reported its safety and tolerability but with varying egg reduction rate (ERR) and cure rate (CR) [21–25]. The most frequently reported adverse events (AEs) include abdominal pain, vomiting, nausea, dizziness, and diarrhea. The type and frequency of treatment-related AEs vary among populations due to genetic differences, nutritional status, age, intensity of infection, and other factors [17–20].

Preventive chemotherapy with single-dose PZQ is crucial to control and eliminate schistosomiasis as a public health problem and to fulfill the WHO strategy—Ending the Neglect to Attain the Sustainable Development Goals: A Road Map for Neglected Tropical Diseases 2021–2030 [26]. Achieving this goal relies, among others, on drug effectiveness and scaling up the program to involve all at-risk groups since untreated infected individuals can serve as parasite reservoirs for continued transmission in the community. The recent WHO guideline recommends the inclusion of pre-SAC in mass PZQ administration starting from the age of 2 years based on the efficacy and safety evidence reported from SAC and adult studies [1]. In the absence of other treatment alternatives, a single dose of PZQ of 40 mg/kg, which is recommended by the WHO for *S. mansoni* infections in SAC, is suggested for pre-SAC with proper efficacy and safety monitoring [22, 27]. Establishing the safety and efficacy of PZQ in pre-SAC children is urgently needed to inform the national NTD program and policymakers before nationwide scaling up of the intervention in this vulnerable age group. Therefore, the present study investigated the efficacy and safety of PZQ among *S. mansoni*-infected pre-SAC in southern Ethiopia.

## Methods

### Study area, setting, and design

A facility-based efficacy and safety surveillance study was conducted among pre-SAC (children aged 4–7 years) who were identified positive for *S. mansoni* infection during the baseline screening using Kato–Katz for *S. mansoni* infection in Hawella Tulla district, Sidama Region, southern Ethiopia. The study was conducted during August to December 2021 at Bushulo Health Center with comprehensive pediatric healthcare services, including laboratory facility enrolling cases linked from three catchment villages/*kebeles* (lowest administrative units in Ethiopia). The villages were Tullo, Finchawa, and Chefe Kotijebesa. According to the results of NTD mapping done in Ethiopia [28], these villages were categorized as high schistosomiasis prevalent areas.

The sample size based on anticipated ERR was estimated using G* power sample size calculation software. The assumptions used from a previous study [29] conducted among SAC were arithmetic mean (AM) ± standard deviation (SD) of egg count before and after treatment being 365.06 ± 437.8 and 78.6 ± 65.6 respectively, power of 0.9, alpha (α) of 0.05, effect size of 0.2, and within-subject correlation of 0.5. The smaller effect size of 0.2 was considered to get a larger sample size, and the total sample size was 265. However, during the baseline screening for *S. mansoni* infection, 241 children tested positive for *S. mansoni* and constituted the final sample.

### Study population

The study population were pre-SAC diagnosed with *S. mansoni* infection. The eligibility criteria for enrollment were being *S. mansoni*-infected aged 4–7 years, living in the study area permanently, and whose parents or primary caregivers gave written consent. Pre-SAC with known chronic medical conditions confirmed by the study physician on the day of the treatment were excluded from the study. Children who received PZQ treatment within the past 4 weeks and those who participated in any other drug trial during the data collection period were excluded to avoid the impact of other medications on the study. Except for one child who received PZQ before enrollment, all *S. mansoni*-infected pre-SAC from the three villages were enrolled for PZQ efficacy and safety evaluation.

### Diagnosis, treatment, and follow-up

The study employed a community-based recruitment strategy that involved screening of pre-SAC at households in the catchment area of the health center. A total of 1683 pre-SAC (4 to 7 years old) residing at three study villages in the Hawella Tulla district of southern Ethiopia were screened for *S. mansoni* infection using the Kato–Katz technique. *S. mansoni*-infected pre-SAC were linked to the health facility to get treatment. Children diagnosed with *S. mansoni* infection (*n* = 241) were recruited and enrolled in this safety and efficacy surveillance study. After recording baseline sociodemographic data including age, sex, and medical history including pre-existing clinical symptoms, and comorbidities, concomitant medications, participants received a single dose of PZQ (40 mg/kg) as recommended by WHO [30]. Praziquantel 600 mg tablets (Batch M00761, Merck KGaA, Darmstadt, Germany) were obtained from the Ethiopian NTD control program. After meal intake, the treating physician gave each child the appropriate dosage calculated based on the child’s body weight. The tablet was crushed with a mortar and pestle, and the powder was mixed and suspended in 10 ml of syrup-flavored water to mask the bitter taste. Crushing of the tablets avoids the risk of choking, which is a potential adverse event in pre-SAC and helpful to make suspension with syrup-flavored water [31]. The appropriate 40 mg/kg PZQ treatment was given to each participant through direct observed therapy by a trained nurse who prepared the medication. Study participants were followed for 8 days to monitor any treatment-associated adverse events, and efficacy was monitored at 4 weeks post-treatment as described previously [17, 20, 30, 32].

### Stool exam using Kato–Katz technique

A single fresh stool sample was collected both at the baseline (pre-treatment) for diagnosis 4 weeks after drug administration (28 days) for monitoring drug efficacy in terms of ERR and CR following the WHO guideline for assessing the efficacy of anthelminthic drugs against schistosomiasis [30]. The stool samples were assessed through duplicate Kato–Katz thick smears (standard template of 41.7 mg) and *S. mansoni* eggs per gram of stool (EPG) was calculated by multiplying the mean egg count by a constant factor of 24 [33]. *S. mansoni* infection intensities were classified according to light infection (EPG < 100), moderate infection (EPG 100–399), and heavy infection intensities (EPG ≥ 400) following the WHO guideline, as per the recommendation of the WHO [30].

### Assessment of treatment-associated adverse events

Study participants were closely monitored for any treatment-associated AEs, including gastrointestinal, neurological, and dermatological symptoms within 4, 24, and 192 h of drug administration. The intensities of the reported AEs were graded as mild, moderate, and severe. Severity grading of treatment-associated AEs was done using the following Common Terminology Criteria for Adverse Events (CTCAE) Version 5.0 [34]:

- Grade 1 (mile): asymptomatic or mild symptoms that require clinical or diagnostic observations only, with no indication of intervention.
- Grade 2 (moderate): moderate AEs limiting age-appropriate Instrumental Activities of Daily Living (ADL); minimal, local, or non-invasive interventions indicated.
- Grade 3 (severe): severe or medically significant AEs not immediately life-threatening but that are disabling and/or limiting self-care ADL. Hospitalization or prolongation of hospitalization indicated.
- Grade 4 (serious)—comprises life-threatening consequences with an indication of urgent intervention.
- Grade 5—death related to AE.

Before the commencement of the study, referral arrangements to a health facility with specialized pediatric care were in place for study participants with definite or suspected serious AEs (any event > grade 2) for further evaluation.

### Study outcomes

The primary study outcome was efficacy (ERR and CR) based on the thick smear Kato–Katz method at 4 weeks post-treatment. The ERR was calculated as 100 times [1 − (Arithmetic mean egg counts at follow-up/Arithmetic mean egg counts at baseline)] per the recommendation of the WHO [30]. The CR was determined as the proportion of infected (egg-positive) children before treatment who became egg-free (egg-negative) at 4 weeks post-treatment. The secondary outcome was treatment-associated AEs experienced or reported within 8 days of drug administration. The major exposure variable was PZQ treatment administered in a single dose of 40 mg/kg. Other covariates for efficacy and safety included age, sex, and co-infection with the three common STHs: hookworm, *Ascaris lumbricoides*, and *Trichuris trichuria*.

### Statistical analysis

All data were recorded on standard record forms, entered the RedCap database, and exported to an Excel file for cleaning. The data analysis was done using Stata version 14 (StataCorp LLC, College Station, Texas, US). Descriptive analyses were done by calculating frequencies and percentages for categorical variables, mean with standard deviations and medians with interquartile ranges for continuous variables. Associations between cure rates and predictor variables were analyzed using binary logistic regression. Predictors of the cure rate for *S. mansoni* infection were analyzed using univariate followed by multivariate logistic regression.

All predictor variables in the univariate analyses were entered into the multivariate model. Adjusted odds ratios (AORs) with a 95% confidence interval (CIs) were reported based on the multivariable logistic regression model. Safety data were analyzed using descriptive statistics and presented in tables and graphs. The log-binomial regression was used to analyze the associations between having any AEs or not with predictor variables. Variables with a *p* value ≤ 0.25 on the crude analysis were included in the multivariable log-binomial regression model. Adjusted risk ratios (ARRs) with 95% confidence intervals (CIs) were reported based on the multivariable log-binomial model. A *p* value ≤ 0.05 was considered statistically significant.

## Results

### Sociodemographic and baseline characteristics

**Fig. 1:**
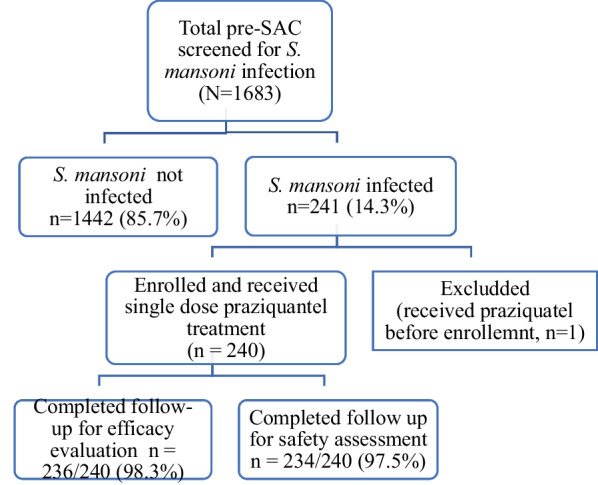
Study flow chart of study participant pre-screening, recruitment, and follow-up among pre-school children

A total of 1683 pre-SAC were screened for *S. mansoni* infection, and 241 (14.3%) had detectable *S. mansoni* infection. Infected children (*n* = 240) were enrolled in this safety and efficacy surveillance study and one child who had received PZQ treatment before enrollment was excluded from the study. Study participants received a single dose of 40 mg/kg PZQ. They were actively followed for 1 week to document any treatment-associated adverse event (safety) and for 28 days to assess the CR and ERR (efficacy). Complete data for efficacy and safety were available from 236 and 234 respectively. Two children withdrew from the study based on the informed decision of their parents. Two pre-SACs from efficacy evaluation and four pre-SACs from safety assessments were lost to follow-up. Figure 1 shows the study flow chart. Table 1 presents the sociodemographic characteristics of the study participants.

**Table 1:** Sociodemographic characteristics of the study participants

Characteristics		*N* (%)
Age (years)	Mean ± SD	5.99 ± 1.03
Age groups	4 to < 6 years	165 (69.9%)
	≥ 6 years	71 (30.1%)
Sex	Male	140 (59.3%)
	Female	96 (40.7%)
District	Tullo	160 (67.8%)
	Finchawa	45 (19.1%)
	Chefe Kotijebesa	31 (13.1%)

N: Total number of participants within each category; SD: standard deviation

### Egg reduction rate and cure rate

The pre-treatment egg count among the pre-SAC ranged from 12 to 2560 EPG (median = 72 EPG; IQR = 24–228 EPG). The overall ERR (93.3%) was above the WHO reference threshold for efficacy regardless of sex, age category, status of STH co-infection or pre-treatment *S. mansoni* infection intensity (Table 2).

**Table 2:** Praziquantel cure rate and egg reduction rate stratified by sex, age category, infection intensity, and co-infection status among *S. mansoni*-infected pre-school children

Variables		Pre-treatment egg count per gram of stool	Cure status			Egg reduction rate (ERR)	WHO reference threshold for ERR [30]
		Median (IQR)	Cured, *n* (%)	Not Cured, *n* (%)	*P*		
Overall		72 (24–228)	201 (85.2%)	35 (14.8%)		93.3%	≥ 90
Sex	Female	84 (24–216)	82 (85.4%)	14 (14.6%)	0.92	92.0%	
	Male	72 (24–231)	119 (85.0%)	21 (15.0%)		94.5%	
Age category	< 6 years	72 (24–168)	61 (85.9%)	10 (14.1%)	0.83	92.5%	
	≥ 6 years	72 (24–240)	140 (84.9%)	25 (15.2%)		93.7%	
Infection intensity at baseline	Light	24 (12–60)	136 (100%)	–	< 0.001	100.0%	
	Moderate	186 (132–288)	55 (83.3%)	11 (16.7%)		93.2%	
	Heavy	816 (627–1242)	10 (29.4%)	24 (70.6%)		92.0%	
STH co-infection	No	60 (24–192)	143 (86.1%)	23 (13.9%)	0.42	92.2%	
	Yes	96 (48–288)	58 (82.9%)	12 (17.1%)		95.8%	

IQR = Interquartile range; SD = Standard deviations; STH = soil-transmitted helminths

Of the 236 pre-SAC, 201 children became egg-free at 4 weeks post-treatment. The overall cure rate was 85.2% (95% CI = 80.0%, 89.5%). There was no significant difference in cure rate between females and males, age categories, or STH co-infection status. However, the cure rate was significantly different based on pre-treatment infection intensity, 100% among children with light infection intensity compared to those with moderate (83.3%) or heavy infections (29.4%).

### Pre- and post-treatment *S. mansoni* infection intensity

All participants with light infection intensity showed 100% parasitological cure, while pre-treatment moderate infection intensity progressed to heavy infection intensity after treatment in one participant (1.5%). No persistent heavy infection intensity following PZQ treatment among pre-SAC was observed (Table 3).

**Table 3:** Cure rate stratified by the pre-treatment and post-treatment infection intensity among *S. mansoni*-infected pre-school children

Pre-treatment infection intensity	*N* (%)	Post-treatment	*N* (%)
Light infection	136 (57.6%)	Light	0
		Moderate	0
		Heavy	0
		Cured	136 (100%)
Moderate infection	66 (28.0%)	Light	7 (10.6%)
		Moderate	3 (4.6%)
		Heavy	1 (1.5%)
		Cured	55 (83.3%)
Heavy infection	34 (14.4%)	Light	12 (35.3%)
		Moderate	7 (20.6%)
		Heavy	0
		Cured	15 (44.1%)

N: Total number of participants within each category; CR: Cure rate

### Predictors of parasitological cure rate

Predictors of cure rate were analyzed using univariate followed by multivariate logistic regression. Sociodemographic characteristics, including age, sex, STH co-infection, and baseline egg count in hundred, were analyzed in the univariate and multivariable regression model. An increase of 100 in baseline egg count resulted in a 26% (95% CI: 17%, 34%) reduction in the odds of cure. None of the other variables had a significant association with the odds of cure (Table 4).

**Table 4:** Predictors of parasitological cure at week four post praziquantel treatment among pre-school children infected with *S. mansoni*

Variable	Category	*S. mansoni* infection status after treatment		COR (95%Cl)	*p* value	AOR (95% CI)	*p* value
		Cured	Not cured				
Age groups (years)	4 to < 6 years	61 (85.9%)	10 (14.1%)	1.0		1.0	
	≥ 6 years	140 (84.9%)	25 (15.2%)	1.08 (0.49, 2.41)	0.8	0.65 (0.27, 1.58)	0.3
Sex	Female	82 (85.4%)	14 (14.6%)	1.0		1.0	
	Male	119 (85.0%)	21 (15.0%)	0.97 (0.47, 2.01)	0.9	0.93 (0 .39, 2.18)	0.9
Infection status	*S. mansoni* only infected	143 (86.1%)	23 (13.9)	1.0		1.0	
	*S. mansoni* and STH infected	58 (82.9%)	12 (17.1%)	0 .78 (0.36, 1.67)	0.5	0 .88 (0.36, 2.13)	0.8
Baseline egg count in hundreds		201 (85.2%)	35 (14.8%)	0.75 (0.67, 0.84)	< 0.001	0.74 (0.66, 0.83)	< 0.001

COR: Crude odds ratio; AOR: Adjusted odds ratio; CI: confidence interval; STH: Soil-transmitted helminths

### Incidence and type of treatment‐associated adverse events

Two hundred thirty-four participants completed the 8-day post-treatment safety follow-up period, and 54 reported experiencing 169 treatment-associated AEs. The overall incidence of experiencing at least one type of treatment-associated AEs was 23.1% (95% CI: 18.0–29.0%). Among the participants who experienced AEs, 33 (61.1%) experienced three or more types of AEs, 15 (27.8%) experienced two types of AEs, and the remaining 6 (11.1%) experienced one type of AE. Of the 169 AEs that occurred after treatment, 110 (65.1%) of the AEs occurred within 4 h of receiving treatment, and 59 (34.9%) occurred during day 2 to 8 follow-up period (Table 5).

**Table 5:** Proportion of treatment-associated adverse events stratified by type and time

Type of AEs	*N*	Day1 at 4 h [*N* (%)]	Day2 at 24 h [*N* (%)]	Day 8 at 192 h [*N* (%)]
Stomachache	27	19 (70.4)	8 (29.6)	
Diarrhea	19	10 (52.6)	7 (36.8)	2 (10.5)
Nausea	18	12 (66.7)	4 (22.2)	2 (11.1)
Drowsiness	17	9 (52.9)	8 (47.1)	
Vomiting	16	10 (62.5)	4 (25.0)	2 (12.5)
Dizziness	15	11 (73.3)	4 (26.7)	
Fever	17	12 (70.6)	5 (29.4)	
Loss of appetite	14	9 (64.3)	5 (35.7)	
Headache	13	8 (61.5)	5 (38.5)	
Cough	3	2 (66.7)	1 (33.3)	
Confusion	5	5 (100.0)		
Itching	3	2 (66.7)		1 (33.3)
Other symptoms	2	1 (50.0)	1 (50.0)	
Total	169	110 (65.1)	52 (30.8)	7 (4.1)

AEs: adverse events; N: total number of AEs; h: hours

**Fig. 2:**
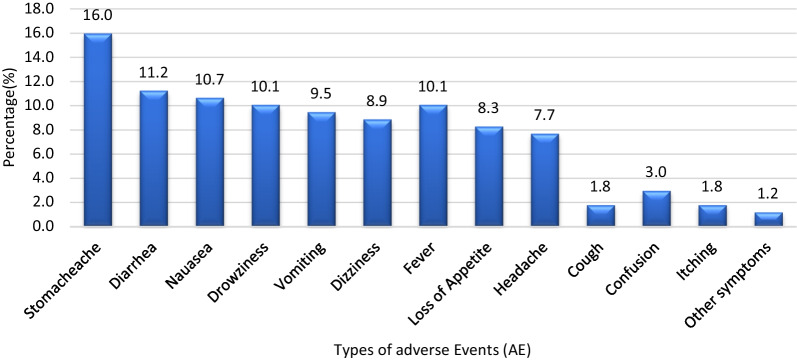
Proportion of praziquantel treatment adverse events over the 8-day follow-up period

Stomachache (16.0%), diarrhea (11.2%), nausea (10.7%), drowsiness and fever (10.1%), vomiting (9.5%), headache (7.7%), dizziness (8.9%), and loss of appetite (8.3%) were the most common AEs following PZQ treatment among *S. mansoni*-infected pre-SAC. The least reported AEs were confusion (3.0%), cough and itching (1.8%), and other symptoms (1.2%) (Fig. 2).

### Severity grading of adverse events

Out of the total 169 treatment-associated AEs, 88.8% (*n* = 150) were mild, 10.7% (*n* = 18) were moderate, and only 0.6% (*n* = 1) were severe. Most of the AEs were transient and resolved within 2 days of post-treatment. No life-threatening AE that required hospitalization (Grade 4) was reported (Table 6).

**Table 6:** Severity grading of reported adverse events following praziquantel treatment among *S. mansoni*-infected pre-school children

Type of AEs	Total number of AEs	Grade1 (Mild)	Grade2 (Moderate)	Grade3 (Severe)
Stomachache	27	20 (74.1%)	6 (22.2%)	1 (3.7%)
Diarrhea	19	17 (89.5%)	2 (10.5%)	
Nausea	18	16 (88.9%)	2 (11.1%)	
Drowsiness	17	15 (88.2%)	2 (11.8%)	
Fever	17	16 (94.1%)	1 (5.9%)	
Vomiting	16	13 (81.2%)	3 (18.8%)	
Dizziness	15	13 (86.7%)	2 (13.3%)	
Loss of appetite	14	14 (100%)		
Headache	13	13 (100%)		
Confusion	5	5 (100%)		
Cough	3	3 (100%)		
Itching	3	3 (100%)		
Other symptoms	2	2 (100%)		
Total	169	150 (88.8%)	18 (10.6%)	1 (0.6%)

N: Total number of AEs within each type; AEs: Adverse events

### Predictors of treatment-associated adverse events

In multivariable log-binomial regression, residing in Tullo village (ARR = 8.8, 95% CI: 1.28, 60.93) and pre-treatment moderate infection intensity (ARR = 3.2, 95% CI: 1.69, 6.14) and heavy infection intensity (ARR = 6.5, 95% CI: 3.6, 11.52) were significant predictors of PZQ treatment AEs (Table 7). The distribution of pre-treatment heavy *S. mansoni* infection intensity varied greatly between villages, being higher in Tullo village (18%) followed by Chefe Kotijebesa (10%), and Finchawa village (7%).

**Table 7:** Predictors of adverse events following praziquantel treatment among pre-school children infected with *S. mansoni*

Variable		AEs after treatment		CRR (95%CI)	*p* value	ARR (95% CI)	*p* value
		AEs	No AEs				
Age groups (years)	4 to < 6 years	21 (22.3)	73 (77.7)	1.0		–	
	≥ 6 years	33 (23.6)	107 (76.4)	1.1 (0.65,1.71)	0.8	–	
Sex	Female	18 (18.8)	78 (81.3)	1.0		1.0	
	Male	36 (26.1)	102 (73.9)	1.4 (0.84, 2.30)	0.2	1.2 (0.81, 1.73)	0.4
District	Chefe Kotijebesa	1 (3.2)	30 (96.8)	1.0		1.0	
	Tullo	49 (30.6)	111 (69.4)	9.5 (1.36, 66.18)	0.02	8.8 (1.28, 60.93)	0.03
	Finchawa	4 (9.3)	39 (90.7)	2.9 (0.34, 24.55)	0.3	4.4 (0.54, 35.31)	0.2
Infection status	*S. mansoni* only infected	35 (21.3)	129 (78.7)	1.0		1.0	
	*S. mansoni* + STH infected	19 (27.1)	51 (72.9)	1.3 (0 .78, 2.06)	0.3	–	
Baseline infection intensity	Light	12 (9.0)	122 (91.0)	1.0			
	Moderate	19 (28.8)	47 (71.2)	3.2 (1.66, 6.22)	0.001	3.2 (1.69, 6.14)	< 0.001
	Heavy	23 (67.7)	11 (32.4)	7.6 (4.20, 13.60)	< 0.001	6.5 (3.62, 11.52)	< 0.001
Praziquantel dose	≤ 1.5 tabs	24 (22.6)	82 (77.4)	1.0			
	**> 1.5 tabs**	**30 (23.4)**	**98 (76.6)**	**1.0 (0.65, 1.66)**	**0.9**	**–**	

CRR: Crude risk ratio, ARR: Adjusted risk ratio, CI: confidence interval

## Discussion

In this study, we assessed the safety, tolerability, and effectiveness of single-dose PZQ for the treatment of *S. mansoni* infection among pre-SAC in southern Ethiopia. The type, incidence, and severity grading of treatment-associated AEs that occurred within a week after receiving treatment were assessed. Reported post-treatment AEs were cross-checked and verified with any existing symptoms or clinical conditions before receiving treatment, and efficacy was measured in terms of CRs and ERR following the WHO guideline for assessing the efficacy of anthelminthic drugs against schistosomiasis [30].

Our study had several notable findings. First, the overall PZQ treatment showed satisfactory ERR (93.3%) and CR (85.2%) against *S. mansoni* infection among pre-SAC; Second, pre-treatment infection intensity was a significant predictor of CR. While all children with light and 83.3% with moderate infection intensity were cured, only one-third of those with heavy infection intensity were cured. A higher baseline egg count was associated with reduced odds of cure. Third, overall, PZQ treatment was tolerated, and about one-fifth of pre-SAC (23.1%) experienced at least one type of treatment-associated mild-to-moderate grade AEs during the first 2 days of receiving treatment. Stomachache, diarrhea, and nausea were the most common AEs. The reported AEs were transient, resolving within a week. Fourth, pre-treatment moderate-to-heavy infection intensity was significantly associated with an increased risk of experiencing treatment-associated AEs. No significant effect of sex, age, or STH co-infection status on the safety and efficacy of PZQ was observed.

Monitoring efficacy after multiple rounds of MDA is recommended since repeated drug exposure may result in parasite tolerability and resistance [35]. Our finding indicates that single-dose PZQ significantly reduced *S. mansoni* infection among pre-SAC, and the overall ERR of 93.3% was above the 90% threshold for optimal PZQ efficacy set by the WHO [30]. Previous studies of single-dose PZQ (40 mg/kg) given to pre-SAC reported varying CR and ERR [23]. A randomized controlled trial conducted in southern Côte d’Ivoire to compare a single dose of 20 mg/kg, 40 mg/kg, and 60 mg/kg PZQ reported the best cure (72%) with 40 mg/kg among pre-SAC [36]. A higher CR and ERR in pre-SAC from Sudan (geometric mean egg reduction rates ranging from 96.4% to 99.4%) [37] and from Eastern Ethiopia (96.4% CR, 99.4% ERR) were reported [25]. On the other hand, a considerably low CR (50.6%) and a geometric mean ERR of 66.7% at 6 weeks post-treatment from Niger were reported [38]. Nevertheless, the observed CR and ERR in pre-SAC in our study are in line with a recent report conducted among *S. mansoni*-infected SAC from the same study area (89.1% CR, 93.7% ERR) [20] and a systematic review of other studies from Ethiopia (pooled CR = 89.2% (95% CI: 85.4–93.1), mean ERR = 90.2%) [39].

The difference in the PZQ treatment outcome between populations could be due to variability in infection intensity prevalence in different geo-locations, and the follow-up time in the study setting might contribute to the difference in ERR among pre-SAC infected with *S. mansoni*. Furthermore, recent studies highlight the importance of genetic variation for PZQ disposition [19, 40], but the impact of genetic variations on PZQ treatment outcomes needs further investigation. Despite the relatively lower CR of pre-SAC with moderate-to-heavy intensity infection groups than the light-intensity infection, the moderate-to-heavy intensity showed satisfactory ERR. This finding indicates that PZQ could reduce morbidities associated with moderate-to-heavy intensity infections among pre-SAC in hyper-endemic communities. This might be related to the paralyzing effect of PZQ on the parasite fecundity, which, in turn, results in low egg count post-treatment, as reported in a previous study [41].

In the present study, even though PZQ showed a high CR against *S. mansoni* infection, about 14.8% of the study participants were not cured of the infection 4 weeks after treatment. The CR among moderately to heavily infected pre-SAC was significantly lower (29.3%) than those who had light (100%) or moderate (83.3%) infection intensity. An increase of 100 in baseline egg count decreased the odds of cure by 26%. Several studies reported significantly reduced CR among moderate-to-heavy infected children than light infections [18, 20, 32]. This could be attributed to the poor efficacy of PZQ against the immature/juvenile stage of the parasites [11, 18]. Repeated infections leading to suppression of the immune response resulting in the survival of the adult worms to favor the spread of infection might also be responsible for the ineffectiveness of PZQ in curing all study participants. This highlights the need to integrate other intervention measures besides MDA, as WHO recommended, to enhance the achievement of the elimination target [1]. Although the observed ERR and CR in the present study indicate that PZQ is effective against *S. mansoni* infection to reduce morbidity among pre-SAC in endemic communities, the search for new anti-schistosome drugs or other alternative treatment strategies, such as drug combinations targeting the different developmental stages of the parasites, is imperative to eliminate the diseases [18, 42].

The present study showed that a single dose of PZQ (40 mg/kg) is tolerable among *S. mansoni*-infected pre-SAC. The overall cumulative incidence of experiencing at least one type of AE among pre-SAC was 23.1% within 8 days of PZQ administration. A recent study among *S. mansoni*-infected SAC (17.0%) from the same study area in Ethiopia and another study in Rwanda (20.6%) revealed a slightly lower incidence of AE compared to our study in pre-SAC [17, 20]. Our study finding aligns with previous reports of a higher incidence rate of AEs in pre-SAC than SAC [43]. The most common AEs observed after receiving PZQ were gastrointestinal disorders. Our finding aligns with previous reports in pre-SAC from elsewhere [38]. The difference in reported AE incidence may be due to variations in intensity of infections and parasite transmission. Genetic differences in the study population, physiological, nutritional, follow-up period, and other environmental factors may also cause variability in treatment outcomes [21, 43]. The difference in AE incidence in various population segments indicates the need for safety monitoring since results from SAC and adult studies may not apply to pre-SAC.

Fifty-four pre-SAC reported a total number of 169 AEs during the follow-up period. Of the total treatment-associated AEs, 65.1% occurred within 4 h of drug intake, and the rest occurred between days 2 to 8 post-drug administration. The most common AEs reported in this study were stomachache, nausea, diarrhea, drowsiness, fever, and vomiting. This observation is similar to previous studies conducted among children aged 2 to 15 in Angola [43] and SAC in Ethiopia [29]. Cough and rash were the least reported AEs in the present study. Almost all the observed AEs were mild and self-limiting, which agrees with reports from studies among SAC in Rwanda [17], Ethiopia [20], and another previous study [44].

The present study showed residing in Tullo village with a higher moderate-to-heavy *S. mansoni* infection intensity was a significant predictor of AEs. The highest incidence of AEs (30.6%) was observed at Tullo village, followed by 9.3% at Finchawa village and 3.2% at Chefe Kotijebesa village. Residing in Tullo village increased the risk of experiencing at least one AE by 20% compared to living in Chefe Kotijebesa village. The increased risk of AEs in Tullo village children could be due to the high number of cases with heavy-intensity infection in this village. The village is closer to the infested water body bordering Lake Hawassa than the other study villages. Pre-SAC with heavy baseline infection intensity had a 6.5 times increased risk of experiencing an AE, and those with moderate infection intensity experienced a 3.2 times increased risk compared to those with light infection intensity. A similar finding was reported by a study conducted in the same area among SAC [45]. Thus, safety monitoring of PZQ administration to pre-SAC living in hyper-endemic villages and high pre-treatment infection intensity remains crucial.

Following the WHO guideline to assess anthelminthic drug efficacy [30], we used the Kato–Katz technique to determine CR and ERR and compare them with the threshold set in the guideline to conclude PZQ effectiveness. The Kato–Katz technique can accurately detect schistosome eggs in stool in high-intensity infection settings to diagnose the disease and conduct infection-intensity assessment, and program evaluation. However, in low-intensity infection settings, the Kato–Katz technique is less sensitive and may result in an overestimation of the efficacy. Hence, using this technique in our study can be considered a limitation. Nevertheless, our surveillance study was conducted in a moderate-to-high-intensity infection area [8], and the CR and ERR observed in our study may accurately estimate PZQ efficacy. On the other hand, point-of-care urine circulating cathodic antigen (POC-CCA), the other alternative diagnostic method, is not yet approved by WHO to assess anthelmintic drug efficacy, and it requires further evaluation, including post-treatment CCA clearance time in different age groups and epidemiological settings [46, 47]. Findings from this study provide valuable evidence on the safety and efficacy of single-dose PZQ treatment among *S. mansoni*-infected pre-SAC for the national NTD program in Ethiopia and the SSA region that follows the WHO guidelines and NTD control strategies.

## Conclusions

Single-dose PZQ (40 mg/kg) administered to *S. mansoni*-infected pre-SAC is effective in curing light-to-moderate infection intensities and reducing morbidity in heavy infections. The treatment is tolerable, and associated AEs are mostly mild-to-moderate and transient resolving within a week. Pre-treatment moderate-to-heavy intensity infection significantly predicts failure to cure and experience treatment-associated adverse events. The lack of cure in 35 (14.8%) and adverse events in one-fifth (23.1%) of *S. mansoni*-infected pre-SAC highlights the need for consistent and close monitoring of the efficacy and safety of the drug, especially in high-transmission and high infection-intensity endemic area. We advocate the inclusion of pre-SAC in the national deworming program and other disease control intervention measures to accelerate the achievement of eliminating schistosomiasis as a public health problem by 2030. Schistosomiasis control program could be extended to pre-SAC using crushed PZQ tablets before the availability of a pediatric formulation.

## Data Availability

All data presented in this study are contained within the manuscript.

## Abbreviations

CR: Cure rate
ERR: Egg reduction rate
MDA: Mass drug administration
PZQ: Praziquantel
Pre-SAC: Pre-school age children
STH: Soil-transmitted helminths
SSA: Sub-Saharan Africa
